# Effects of low-purine diet supplemented with *Sida acuta* Burm. f. on growth performance, purine deposition, and biomolecules in slow-growing chickens

**DOI:** 10.5713/ab.24.0798

**Published:** 2025-02-27

**Authors:** Orapin Jantasaeng, Michel Jacques Duclos, Kanjana Thumanu, Supattra Okrathok, Sutisa Khempaka

**Affiliations:** 1 School of Animal Technology and Innovation, Institute of Agricultural Technology, Suranaree University of Technology, Nakhon Ratchasima, Thailand; 2 Avian Biology and Poultry Research, French National Institute for Agriculture, Food, and Environment, Université de Tours, Nouzilly, France; 3 Synchrotron Light Research Institute (Public Organization), Nakhon Ratchasima, Thailand

**Keywords:** Growth Performance, Low-purine Diet, Meat Quality, *Sida acuta* Burm. f., Slow-growing Chicken

## Abstract

**Objective:**

This study investigated the effects of a low-purine (Pu) diet supplemented with *Sida acuta* Burm. f. (SA) on growth performance, serum uric acid (SUA), and meat quality, including chemical composition, biomolecules, and purine deposition, in slow-growing Korat chickens (KRC).

**Methods:**

A total of 480 mixed-sex one-d-old KRC were randomly allocated into five groups with six replicates each (16 chicks/replicate) using a 1+2×2 augmented factorial experiment in a completely randomized design. Five experimental diets were as follows: a control (basal diet), two diets with 30% and 45% lower purine levels than the control (−30% and −45% Pu), each supplemented with two levels of SA (0.3% and 0.6% SA), respectively. After sex determination, all subsequent analyses were conducted exclusively on female birds.

**Results:**

KRC fed a −30% Pu diet supplemented with 0.6% SA showed reduced hypoxanthine and total purine deposition in major breast muscle (*Pectoralis major*) without negative effects on growth performance, SUA levels, meat quality, chemical composition, biomolecules, and protein secondary structures comparable to the control group. Birds fed a −45% Pu diet, supplemented with either 0.3% or 0.6% SA, exhibited impaired growth performance and without significant changes in major breast muscle hypoxanthine or total purine content. Correlation analysis revealed that the −30% Pu diet supplemented with 0.6% SA was positively correlated with reduced hypoxanthine and total purine content, while showing negative correlations with lipid content and β-turn structure.

**Conclusion:**

A 30% reduction in dietary purines combined with 0.6% SA supplementation effectively decreased purine deposition in chicken meat, contributing to the production of healthier chicken meat product. Further studies are needed to investigate the molecular mechanisms and metabolic pathways, which will enable breeders to advance the development of Pu chicken meat production.

## INTRODUCTION

Dietary purines, including nucleotides, nucleosides, and bases (adenine, guanine, hypoxanthine, and xanthine) exist naturally in the diet. These compounds can be acquired both endogenously through the body’s synthesis and exogenously through dietary sources, serving as essential nucleic acids that play crucial roles in various biological cellular processes [1]. However, excessive consumption of purine-rich foods has been reported to significantly affect serum uric acid (SUA) levels in humans, potentially leading to hyperuricemia (HUA) and an increased risk of gout [2]. Nutrition experts recommend that patients with gout limit their consumption of purine-rich foods, particularly those high in uricogenic purines like adenine and hypoxanthine [2]. Therefore, the investigation of low-purine (Pu) foods has become a crucial focus in the dietary management of both diseases.

Korat chicken (KRC), a slow-growing breed developed by crossbreeding male Thai indigenous chicken (Leung Hang Khao) with a female Suranaree University of Technology (SUT) synthetic breeder line [3]. KRC has a firm and chewy texture and is preferred over broiler meat by domestic Thai consumers [3]. It contains a lower level of total purines compared to broiler chickens (181 vs. 196 mg/100 g), though slightly higher than other Thai native slow-growing chickens, such as the Pradoo-Hangdum chicken (142 mg/100 g) [4]. This pattern suggests that genetic factors in slow-growing chicken breeds contribute to lower purine content comparted to commercial broilers. While chicken meat serves as an important source of nutrients and is crucial for a balanced diet, there is limited information on exploring nutritional strategies to manipulate its purine content. Sex differences also play a significant role, as female chickens (particularly Korean native chicken) have been found to accumulate lower levels of purine derivatives (hypoxanthine) than males [5]. Our previous research has already documented that male KRCs fed with Pu diets supplemented with *Sida acuta* Burm. f. (SA) showed no significant changes in major breast muscle purine content [6]. Currently, female KRCs are less favored by farmers due to their slower growth rate comparted to males, despite commanding the same market price [7]. Therefore, promoting the production of female KRC with naturally low purine content could serve dual purposes: offering a healthier meat option while providing farmers with an alternative strategy to enhance competitiveness and economic returns.

The homeostasis of purine nucleotides in cells is regulated through three processes: 1) *de novo* purine synthesis from smaller organic molecules, 2) salvage of preformed free purine bases, and 3) purine uptake from extracellular sources (diet) via salvage metabolic pathways [8]. In animal feed ingredients, purine content varies considerably by source. High-protein ingredients such as meat meal, fish meal, and soybean meal contain elevated purine levels (100 to 200 mg purine/100 g), while plant-based ingredients such as corn gluten meal, corn, cassava, rice bran, and oilseed have substantially lower purine content, generally below 50 mg purine/100 g [9]. Previous research has shown that reducing the purine content in KRC diets by 30% results in a decrease of approximately 8.0% in total purine deposition, without negative effects on growth performance or meat quality [9]. In addition to Pu diets, natural plant flavonoids, such as quercetin and kaempferol, have been extensively studied for their ability to lower SUA [10]. Dietary supplementation with the SA flavonoid combined with Jerusalem artichoke effectively decreased hypoxanthine and total purine accumulation in KRC meat without compromising growth performance, meat quality, or inosine monophosphate (IMP) content [11]. In general, xanthine oxidase (XO) plays a critical role in the purine catabolism pathway, and inhibiting its activity effectively reduces SUA levels. Research conducted in chicken has shown that flavonoids can reduce SUA concentrations by inhibiting XO activity [10]. SA, a medical plant rich in flavonoids, has shown potential to inhibit the XO enzyme activity and consequently reduce SUA levels in chickens [11].

Synchrotron radiation-based Fourier transform infrared (SR-FTIR) microspectroscopy is a powerful technique known for its high sensitivity, rapidity, and non-destructiveness nature, provide valuable insights into the detection of biochemical compounds, such as proteins, lipids, glycogen, and carbohydrates, at the molecular level [12]. This techniques has been successfully applied to monitor biochemical changes in chicken meat, enabling researchers to distinguish between commercial broilers and slow-growing chickens [13]. The application of SR-FTIR could therefore by valuable in monitoring biomolecular changes in KRC meat fed varying levels of Pu diets and natural plant supplements.

Based on the information provided above, we hypothesized that the reduction of purines in the diet, in combination with SA flavonoids, which act as inhibitors of the XO enzyme, could reduce purine accumulation in meat without adversely affecting growth performance, meat quality, or chemical composition of KRC. In addition, SR-FTIR analysis could provide detailed insighted into the biomolecule changes in KRC meat in response to these dietary modifications. Therefore, this study aimed to investigate the effects of SA supplementation on various aspects of female KRC meat, including growth performance, SUA levels, meat quality, chemical composition, purine deposition, and biomolecule profiles.

## MATERIALS AND METHODS

### Ethics statement

The current experiment was approved by the Animal Ethics Committee of Suranaree University of Technology (approval number: SUT-IACUC-012/2020) and adhered to the Ethics of Animal Experimentation of the National Research Council of Thailand.

### Birds, experimental design, and diets

Due to the inability to determine KRC gender at one day after hatching [3], mixed-sex birds were raised in present study throughout experimental period, with sex identification and wing-banded performed at 21 d of age.

A total of 480 mixed-sex one-d-old KRC were obtained from the Poultry Research Unit of the SUT Farm, with an initial body weight (BW) of 42.4±3.25 g (mean±standard deviation). The birds were randomly allotted to five treatments using a 1+2×2 augmented factorial with a completely randomized design (CRD), with each treatment group consisting of six replicates (16 birds/replicate). Five dietary treatments were: 1) control (basal diet), 2) a diet with purine content reduced by 30% compared to the control (−30% Pu) and supplemented with 0.3% SA, 3) a diet with purine content reduced by 30% compared to the control (−30% Pu) and supplemented with 0.6% SA, 4) a diet with purine content reduced by 45% compared to the control (−45% Pu) and supplemented with 0.3% SA, 5) a diet with purine content reduced by 45% compared to the control (−45% Pu) and supplemented with 0.6% SA. All experimental diets were formulated according to the NRC [14], with metabolizable energy (ME) and protein contents aligned with Maliwan [15,16]. To achieve targeted 30% and 45% dietary purine reductions compared to the basal diet, feed ingredients were carefully selected to reduce protein content by 1.5% to 3.0%. Specially, corn gluten meal partially replace soybean meal due to its lower purine content [9], which enabled maintenance of adequate protein levels while successfully reducing overall purine content in the experimental diets. Synthetic amino acids were added to maintain essential amino acid balance in accordance with NRC [14] requirements. The digestible amino acid content of experimental diets was calculated using the coefficient of digestibility for each amino acid in the individual feed ingredients (i.e. corn, soybean meal, and corn distiller’s dried grains with soluble), as reported by Ajinomoto Heartland LLC [17]. In addition, SA was incorporated at 0.3% and 0.6% levels, providing 25 and 50 mg crude flavonoids/kg diet, respectively. The experimental diets were fed across three periods: starter (1 to 21 d), grower (22 to 42 d), and finisher (43 to 63 d). The ingredient compositions of the experimental diets are presented in Table 1, and the calculated and analyzed values of the experimental diets are shown in Table 2.

The birds were housed in a naturally ventilated open-sided barn and raised under consistent environmental and management conditions throughout the experimental period. The birds were vaccinated against Newcastle disease and Infectious Bronchitis at 7 and 21 d of age, respectively, and vaccinated against infectious bursal disease at 14 d of age and Fowl pox disease at 35 d of age. Each pen (2×2 m^2^) was equipped with a tray feeder and drinker during the first 14 d of age. Starting from d 15, a nipple-type drinker line with seven 7 nipples and round-bottomed hanging feeders was used to supply water and feed. Birds were allowed unrestricted access to feed and water throughout the experiment. To maintain brooding heat, an infrared heat lamp bulb (175 W) was used during the first 14 d of age, with one lamp for each pen. A brooding temperature of 35°C was maintained during the first week after hatch and subsequently was gradually reduced by 3°C per week until it reached room temperature. All birds were exposed to a 23 h photoperiod using a fluorescent bulb as the light source.

### Data collection and measurement

*Growth performance*: Mixed-sex KRC were used in this study, as previously described. Growth performance evaluation involved measuring BW and feed intake (FI) per pen at the beginning and end of each experimental period (21, 42 and 63 d of age). These measurements were used to calculate BW gain (BWG), and feed conversion ratio (FCR). Since the primary objective was to reduce purine deposition in female KRC, separate analysis of BW began at 21 d of age when sex identification became possible individual BW measurements were taken at the end of each experimental period (21, 42, and 63 d of age). For female birds (n = 6), mean BW, and BWG were calculated using the average IBW from each mixed-sex pen as the baseline.

*Sampling procedures*: At the end of each period (21, 42, and 63 d of age), blood samples were collected from 30 female birds (six birds/treatment, with one bird/replicate) via the jugular vein and placed in vacuum tubes containing a clot activator. The tubes were centrifuged at 4,000×g at 4°C for 10 min to separate serum. After centrifugation, the serum was aliquoted into individual microcentrifuge tubes and stored at −80°C until further analysis for UA concentration.

On day 63, two set of female birds were selected for different analyses. For the first set, 30 female birds (six birds/treatment, with one bird/replicate) were selected with a BW close to the pen mean and were subjected to a 12 h fasting period with water access, then sacrificed by electric stunning and decapitation, followed by bleeding, scalding, mechanical defeathering, and manual evisceration. The carcasses were then chilled at a temperature of 4°C for a duration of 24 h. Afterwards, the quality of the major breast muscle (*Pectoralis major*) was evaluated.

For the second set, 30 female birds (six birds/treatment, with one bird/replicate) were selected with BW close to the flock mean for analysis purine and its derivatives (nucleotides), chemical, and biomolecule composition in major breast muscle. This selection criterion was based on previous research [18] indicating that purine deposition varies with the final BW of animals, including chickens, where heavier animals tend to deposit more purines. To minimize the confounding effects of BW variation within each treatment groups, birds were selected based on the flock average BW. These birds were subjected to a 12 h fasting period with access to water before being slaughtered by decapitation and bleeding. Following slaughter, samples of major breast muscle from the right side were collected, vacuum-packed, and stored at −80°C for subsequent analysis of purine and nucleotide content. The remaining portions were placed in a zip-lock bag, stored at 4°C for 24 h, and further used for biomolecule analysis conducted using SR-FTIR. Major breast muscle samples from the left side were collected for chemical composition analysis, including dry matter, crude protein, ether extract, and ash.

*Meat quality measurement*: To determine the quality of chicken meat, the collected samples of chicken breasts were analysed. At 45 min and 24 h postmortem, pH values were measured using a MiniScan EZ colorimeter (Hunter, Reston, VA, USA). Calibration of the colorimeter was performed using black and white calibration tiles, according to the standard manual. The “Commission Internationale de l’Eclairage” (CIE), L^*^ (lightness), a^*^ (redness), and b^*^ (yellowness) values were recorded in accordance with the CIE. Each sample was analyzed at three locations on the meat surface [19].

Drip loss: meat samples were cut into 1.3 cm pieces after chilling for 24 h. Then the cut samples were hung inside a chilled storage at 4°C for 24 h. The percentage of drip loss was calculated using the following equation: Drip loss (%) = [(initial weight − final weight) / initial weight] × 100 [20].

Cooking loss: meat samples were weighed and boiled in a water bath in plastic bags until an internal temperature of 80°C was reached. The cooking loss was determined and calculated using the following equation: Cooking loss (%) = [(sample weight before cooking − sample weight after cooking) / sample weight before cooking] × 100 [20].

The shear force was determined using a texture analyzer (TA-XT Plus, Stable Micro System Ltd., Surrey, UK). A sample (2×1.0×1.0 cm^3^) was obtained from paralleled muscular fibers in the medial portion of the breast by using a knife. The shear force values were determined for three strips per sample, and the test speed was set to 2 mm/s. Data were collected and analyzed from the shear force values to obtain the maximum force required to shear each sample, and the results were expressed in kilograms [20].

*Major breast muscle chemical composition*: Major breast muscle samples were collected to assess their chemical composition, which included dry matter, ether extract, and ash, according to the methods of the Association of Official Analytical Chemists [21]. Crude protein (CP) was determined using the Dumas combustion technique with a nitrogen analyzer (Elementar Analysensysteme GmbH, Hanau, Germany), in which L-aspartic acid (Sigma-Aldrich, St Louis, MO, USA) was used as the calibration standard. The CP content was calculated by multiplying the nitrogen content by a factor of 6.25 [22].

*Major breast muscle purine analysis*: The purines (adenine, guanine, hypoxanthine, and xanthine) in major breast muscle were determined using high-performance liquid chromatography (HPLC) following the procedure described by Kaneko et al [23], with some slight modifications. Briefly, major breast muscle samples were minced and weighed (500 mg), hydrolyzed 35% perchloric acid, and then boiled at 95°C for 1 h. The hydrolyzed solution was neutralized, and the supernatant was collected. Purine bases were separated on an ZORBAX SB-C18 column (4.6×250-mm, 3.5-μm particles, Agilent Technologies, Santa Clara, CA, USA) equipped with a HPLC system (HP 1260; Agilent Technologies). The quantities of guanine, adenine, xanthine, and hypoxanthine were determined by comparing their respective peak areas with the corresponding calibration curves of external standards (Sigma-Aldrich Co.). The results are expressed as mg/100 of the sample wet weight. The total purine content is the sum of the adenine, guanine, hypoxanthine, and xanthine contents. Measurements were conducted in duplicate for each sample.

*Major breast muscle nucleotide analysis*: Nucleotide content (IMP and guanosine monophosphate [GMP]) was measured following a method adapted from Katemala et al [13] with slight modifications. Briefly, the extracted nucleotides were analyzed using an HPLC system (HP 1260; Agilent Technologies) equipped with a ZORBAX SB-C18 column (4.6×250-mm, 3.5-μm particles, Agilent Technologies). The mobile phases were as follows: mobile phase A, consisting of 150 mM KH_2_PO_4_ and 150 mM KCl (pH 6.0), and mobile phase B, which was prepared by mixing mobile phase A with 20% acetonitrile, a flow rate was maintained at 0.5 mL/min. The composition of the mobile phase varied as follows during the analysis: initially, 3% B was used for the first 5 min, followed by an increase to 9% B from 5 to 10 min, a further increase to 20% B from 10 to 15 min, and 100% B for 15 to 20 min, and then maintained at 100% B for 15 min. The amount of IMP and GMP were calculated by comparing their respective measurements with external standards (Sigma-Aldrich Co). All samples were subjected to duplicate measurements and the values were averaged to obtain the final results.

*Serum uric acid concentration measurement*: The concentration of UA in serum was determined using a commercially available diagnostic kit (BioSystems S.A. Costa Brava 30, Barcelona, Spain) following the manufacturer’s instructions, which were based on the method originally described by Fossati et al [24].

*SR-FTIR microspectroscopy*: Major breast muscle samples were cross-sectionally cut into 1×1 cm pieces, placed into an aluminum foil block filled with optimal cutting temperature, and snap-frozen in liquid nitrogen. Subsequently, the frozen tissue sections were cut at a 5 μm thickness on a cryostat microtome (AST 500 Semi-automatic Cryostat Microtome) and placed on the barium fluoride (BaF_2_) window (Crystran Ltd., Dorset, UK) and dried in a vacuum chamber for two to three days before the SR-FTIR analysis. Six major breast muscle samples from each group (two muscle sections from each chicken) were subjected to spectral analysis. FTIR spectra were measured at BL4.1 Infrared spectroscopy & Imaging, Synchrotron Light Research Institute using an SR-FTIR spectrometer with a synchrotron light source in the mid-IR region. Spectra were collected on a Bruker FTIR spectrometer (Vertex70; Bruker Optics Ltd., Ettlingen, Germany) coupled to a Bruker Hyperion 2000-IR microscope (Bruker Optik GmbH) with a 36× objective, coupled to an MCT detector cooled with liquid nitrogen, covering a measurement range of 4,000 to 800 cm^−1^. The FTIR spectra were obtained in transmission mode, collecting 64 scans with a 10×10 mm aperture size at a resolution of 6 cm^−1^ over a measurement range of 4,000 to 800 cm^−1^. The raw spectra from each group consisting of 150 spectra (25 spectra×6 chickens) were processed using the OPUS 7.5 software (Bruker Optics Ltd.).

The determination of the average original and secondary derivatives in the important functional group region of SR-FTIR spectra for female KRC major breast muscle using the method described by Suwanvichanee et al [25]. Briefly, the integral areas were determined using second derivative processing at the spectral regions in several regions: 3,000 to 2,800 cm^−1^ (CH stretching of lipids); 1,700 to 1,600 cm^−1^ (amide I), 1,600 to 1,500 cm^−1^ (amide II), 1,450 to 1,390 cm^−1^ (CH binding), 1,320 to 1,220 cm^−1^ (amide III), 1,200 to 900 cm^−1^ (glycogen/carbohydrate) using the OPUS 7.5 software (Bruker Optics Ltd.). The curve fit of amide I (1,700 to 1,600 cm^−1^) provides the estimate of the secondary structure of protein including α-helix (1,644, 1,655 cm^−1^), β-sheet (1,630 cm^−1^), β-turn (1,670 cm^−1^), and antiparallel (1,689 cm^−1^) regions based on 10% Gaussian and Lorentzian functions using the OPUS 7.5 software (Bruker Optics Ltd.).

*Statistical analysis*: Significant difference analysis: all data were analyzed using analysis of variance as a 1+2×2 augmented factorial experiment in the CRD following the general linear model procedure of SAS OnDemand for Academics software (SAS Institute Inc., Cary, NC, USA). The statistical model considered the control, Pu level, SA level, and their interactions. Orthogonal contrasts were applied to compare the control group with the other treatment groups and the interactions between Pu and SA levels. The effects of the experimental diets were determined by orthogonal contrasts 1) control vs. other treatment groups, 2) level of Pu (−30% vs. −45% Pu), 3) level of SA (0.3% vs. 0.6% SA), and 4) interaction Pu×SA. The probability level of orthogonal contrasts was considered statistically significant at p<0.05. In the case of significant contrasts 1) or 4), differences in treatment means were evaluated using Tukey’s multiple comparison test and considered significant at p<0.05. All values in the text and tables are reported as means ± SEM.

Principal Component Analysis: principal component analysis (PCA) was used to identify biochemicals. All spectral data were preprocessed using the Savitzky-Golay algorithm for second-derivative transformations at 13 smoothing points and normalized with extended multiplicative signal correction using Unscrambler X Multivariate Data Analysis software (version 10.4; Camo Analytics, Oslo, Norway). The 150 spectra were averaged into 30 spectra per group and outliers were removed. Subsequently, the obtained data were re-averaged into six spectra per treatment group or cluster using PCA. Bi-plot correlation was used to represent the clustered differentiation of the data, and the related variables were recalculated using a two-dimensional scatter plot of PCA with the predominant spectral range. The high-loading SR-FTIR spectra were selected for multivariate analysis of biomolecules, secondary structure of proteins, chemical composition, purine and nucleotide contents in meat. The relationships between the variables were investigated using PCA biplot correlation.

## RESULTS

### Growth performance

The effects of a Pu diet supplemented with SA on the growth performance of mixed-sex KRC are presented in Table 3. No significant interaction was observed between Pu and SA levels throughout the experimental period. Compared to the control group, significant differences results were found in BW, BWG, and FCR during the starter (1 to 21 d), grower (22 to 42 d), and overall growth periods (1 to 63 d) (p<0.001). During the finisher period (43 to 63 d), only BW showed significant differences among treatments (p<0.001). While SA supplementation had no significant effect on growth performance, the Pu level significantly affected BW, BWG, and FCR during the starter, grower, and overall growth periods (p<0.001). Interestingly, KRC fed with −30% Pu diet supplemented with either 0.3% or 0.6% SA maintained growth performance comparable to the control group. However, birds fed with −45% Pu diet showed significantly lower BW, BWG (p<0.05) and higher FCR (p<0.05) compared to the control group. In female birds (Table 4), similar trends were observed in BWG, suggesting that effect of dietary purine reduction was consistent across both sexes.

### Meat quality and chemical composition

Dietary treatments did not show any significant effects on various parameters such as pH, L*, a*, drip loss, cooking loss, and shear force of female KRC major breast muscle (Table 5). However, a significant difference was observed between the control and other groups for meat color in b* (p<0.001), where dietary low purine supplemented with SA group resulted in an increase in compared to birds fed the control diet (p<0.05). Regarding the chemical composition of the major breast muscle, it was also observed that dietary treatments had no significant effect on dry matter, crude protein, ether extract and ash (Table 5). No interaction was observed between the Pu and SA levels for any meat quality or chemical composition measurements.

### Serum uric acid concentration

The effects of different diets on SUA concentrations in female KRC at 21, 42, and 63 days of age are presented in Table 6. This did not affect the SUA concentration at 21 days of age. However, KRC fed a −45% Pu diet supplemented with 0.6% SA showed a significant decrease (p<0.05) in SUA concentration as compared to the control group at 42 days of age (7.25 vs. 3.48 mg/dL), and a similar trend at 63 days of age (p = 0.065). There were no significant effects of Pu or SA levels, and no interaction was observed between Pu and SA levels at any age.

### Major breast muscle purine and its derivatives deposition

The effects of dietary low purine supplemented with SA on purine and its derivatives deposition in female KRC major breast muscle at 63 days of age are presented in Table 7. An interaction between Pu and SA level was observed for hypoxanthine (p = 0.022) and total purine (p = 0.025) in female KRC major breast muscle. It was found that the major breast muscle from female KRC fed a dietary −30% Pu supplemented with 0.6% SA exhibited significantly lower levels of hypoxanthine and total purine deposition compared to the control group (p<0.05). Meanwhile, no significant effects were observed among the treatments for guanine, adenine, and nucleotides (GMP and IMP) in female KRC major breast muscle. In addition, xanthine was not detected in the major breast muscle of any groups in this study.

### Biomolecules and secondary protein structure changes in Korat chicken major breast muscle using synchrotron radiation-based Fourier transform infrared

**Figure 1:**
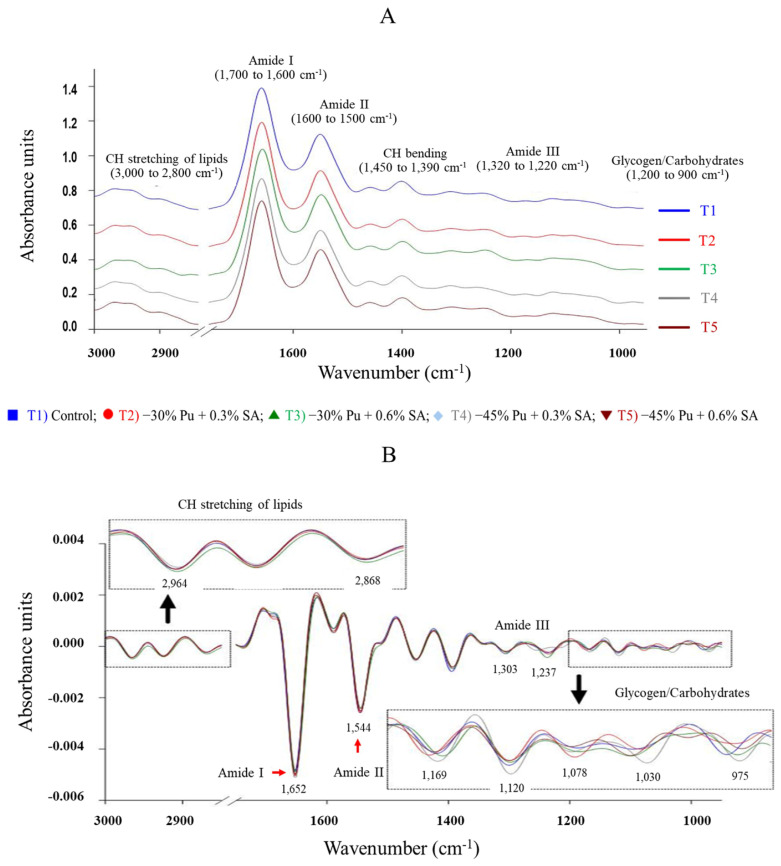
The average synchrotron radiation-Fourier transform infrared (SR-FTIR) spectra of the original (A) and second-derivative (B) spectra from female Korat chickens major breast muscle fed low-purine (Pu) diet supplemented with *Sida acuta* Burm. f (SA). The infrared spectra were detected in the region from 3,000 to 900 cm^−1^, reveal distinct features including CH stretching of lipids, amide I, amide II, CH bending, amide III, and glycogen/carbohydrates, respectively.

Representative SR-FTIR spectra of the average original and secondary derivatives in the important functional group region, spanning wavenumbers from 3,000 to 900 cm^−1^ for female KRC major breast muscle, are presented in Figures 1A, 1B, respectively. Detailed assignments for this region were based on the method described by Suwanvichanee et al [25]. The average second-derivative spectra from the five experimental diet groups revealed distinct differences in the peak heights at 2,964 and 2,868 cm^−1^ representing lipids (shown by FTIR as CH stretching); 1,652 cm^−1^ representing amide I; 1,544 cm^−1^ representing amide II; 1,303 and 1,237 cm^−1^ representing amide III; and 1,169, 1,120, 1,078, 1,030, and 975 cm^−1^ representing glycogen/carbohydrate (Figure 1B).

Integral areas under the peaks were obtained for the lipid, amide I, amide II, CH bending, amide III, and glycogen/carbohydrate regions. The integration percentages for each biomolecule are listed in Table 8. No interaction was observed between Pu and SA levels for any biomolecule. However, significant effects of the Pu level were noted for lipids (p = 0.001), amide I (p = 0.041), amide II (p = 0.037) and glycogen/carbohydrate (p = 0.028) whereas the SA level had a significant effect only on amide III (p = 0.039). Interestingly, major breast muscle from female KRC fed a −30% Pu supplemented with 0.6% SA showed no significant difference as compared to the control group. Whereas a −45% Pu supplemented with 0.3% SA resulted in higher levels of glycogen/carbohydrate than birds fed the control diet (p<0.05).

The secondary structures of the protein in the amide I region, ranging from 1,700 to 1,600 cm^−1^ were determined by curve fitting [25]. The secondary structures of the protein identified include β-sheet at 1,623 cm^−1^ and 1,635 cm^−1^, α-helix at 1,650 cm^−1^ and 1,660 cm^−1^, β-turn at 1,675 cm^−1^, and antiparallel at 1,690 cm^−1^. The percentages of each type of secondary structure curve fitted for female KRC major breast muscle under different dietary treatments are presented in Table 9. No significant effects of dietary treatments were observed on the secondary structure of proteins in the amide I region of female KRC major breast muscle.

### Correlation loading plot of synchrotron radiation-based Fourier transform infrared spectra with biomolecules, secondary structure proteins, purine, and its derivatives deposition from various dietary purine and *Sida acuta* Burm. f. levels

**Figure 2:**
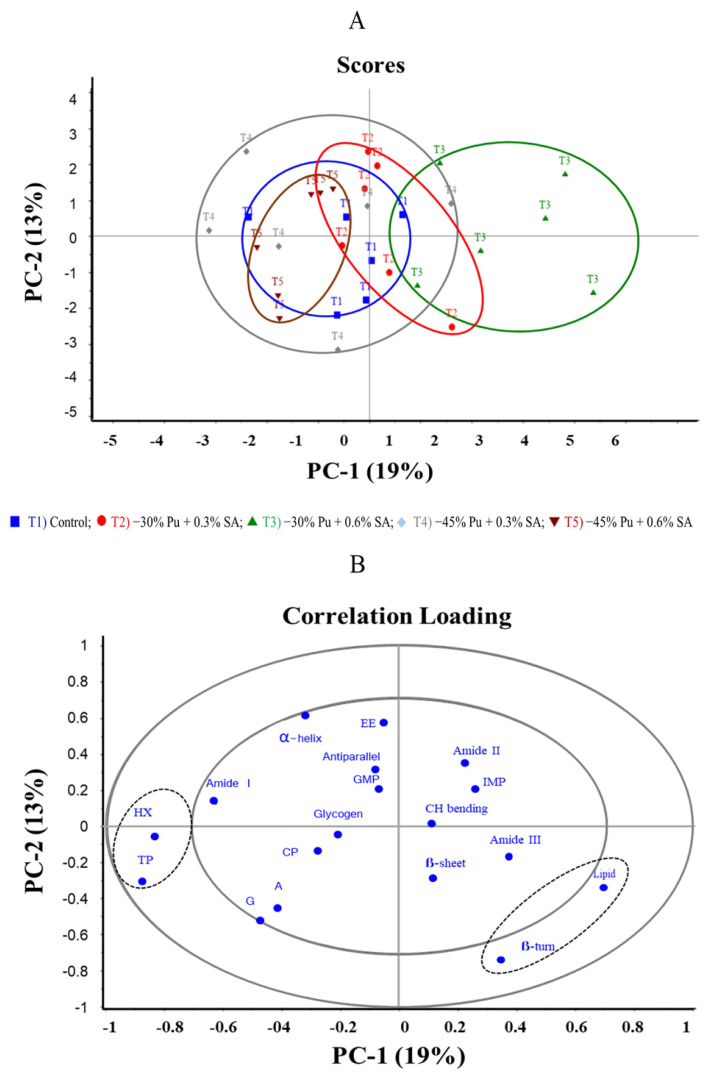
Principal component analysis score plot (A) for PC–1 vs. PC–2 of female Korat chickens major breast muscle fed low-purine (Pu) diet supplemented with *Sida acuta* Burm. f. (SA) and correlation loading plot (B) related to biomolecules, secondary structure of protein, chemical composition, purine and nucleotide contents in meat. A, adenine; G, guanine, Hx, hypoxanthine; TP, total purine; IMP, inosine monophosphate; GMP, guanosine monophosphate; EE, ether extract; CP, crude protein.

The PCA score plot and correlation loading illustrating the relationship between biomolecules, secondary structure of proteins, chemical composition, purine and its derivatives contents in female KRC major breast muscle are shown in Figures 2A, 2B, respectively. A PCA score plot was constructed to assess the correlation of these parameters with PC–1 (19%) and PC–2 (13%) scores, which explained approximately 32% of the total variability. The PCA score plot (Figure 2A) demonstrates distinct separation along PC–1 (horizontal axis) between −30% Pu supplemented with 0.6% SA (T3) and −45% Pu supplemented with 0.6% SA (T5). The remaining treatment groups, including control (T1), −30% Pu supplemented with 0.3% SA (T2), −45% Pu supplemented with 0.3% SA (T4) and −45% Pu supplemented with 0.6% SA (T5), showed overlapping patterns. These overlapping groups displayed a negative correlation with T3 regarding the measured parameters (biomolecules, protein secondary structure, chemical composition, purine and its derivatives) in female KRC major breast muscle.

The PCA correlation loading (Figure 2B), features two ellipses that represent variance: the outer ellipse denotes 100% of the explained variance, whereas the inner ellipse indicates 50%. The variables found between the two ellipses, particularly those near the edges of the outer ellipses, are the most important for differentiation. Variables located in the outer circle region, including total purine, hypoxanthine, lipids, and β-turns, significantly correlated with the experimental groups. Specially, the −30% Pu supplemented with 0.6% SA (T3) was positively correlated with lipids and β-turn but negatively correlated with total purine and hypoxanthine.

## DISCUSSION

Purines in the body originate from endogenous sources (70%) and exogenous sources via dietary intake (30%), with UA being the ultimate product of purine catabolism in both humans and chickens [26]. Chicken meat is highly favored because of its high protein content and affordability; however, it has a high purine content, which may limit its consumption among individuals susceptible to HUA or gout [27]. Our findings revealed that female KRC fed a −30% Pu diet supplemented with SA at a level of 0.6% reduced hypoxanthine and total purine deposition in major breast muscle without adversely affecting growth performance, meat quality (except for meat yellowness), SUA concentration, meat chemical composition, and biomolecule composition as compared to birds fed a control diet. However, a reduction in purine by up to 45%, supplemented with either 0.3% or 0.6% SA, resulted in a decreased growth rate and proved ineffective in reducing purine levels in terms of hypoxanthine and total purine in female KRC major breast muscle, while increasing glycogen/carbohydrate content, compared to birds fed a control diet.

A limitation of this study was the inability to determine KRC gender at hatching, necessitating mixed-sex birds raising throughout the experimental period. Nevertheless, the growth performance pattens in mixed-sex birds paralleled those observed in females. Birds fed a −30% Pu diet supplemented with SA up to 0.6% had no detrimental effects on growth performance (BW, BWG, and FI) compared with the control group throughout the study. This may be attributed to the cells retaining the ability for coordinated purine synthesis between *de novo* biosynthesis and the recycling of free purine bases from dietary purines, or the degradation of genetic material via the salvage pathway [18], ensuring sufficient purine nucleotides for various metabolic processes in the KRC. Regarding SUA, we observed that the growth rate of KRC groups fed with dietary −30% Pu aligns consistent with the SUA concentrations, which exhibited no differences compared to the control. In addition, no significant difference in SUA concentrations was observed between the groups, except for the chicken group aged 42 days receiving the −45% Pu supplemented with 0.6% SA, which had significantly lower SUA concentrations compared to the control. In general, a reduction of SUA is associated with a low purine or protein diet and their metabolism [26], as well as with the balance of amino acids in the diets [28]. Our finding is consistent with previous studies by Tantiyasawasdikul et al [26] and Chaiyasit [9], who reported that reducing purine intake in the diet decreases the production of UA, leading to lower SUA levels in chickens. Additionally, natural flavonoids from plants, particularly those functioning as XO inhibitors, have been extensively studied for their ability to lower UA levels. Ogunmoyole et al [10] reported that treatment with SA extract in rats inhibited UA synthesis, suggesting that XO-inhibitory constituents in SA may down-regulate purine metabolism or reduce UA concentration. This suggests that amino acids are not consumed in excess and that purine nucleotides in the body may also contributed to homeostasis. Furthermore, supplementation of XO inhibitors could potentially be utilized to reduce SUA levels. While the exact cause of this phenomenon remains unclear; it is known that SUA levels can be influenced by various factors including age, diet and species [28].

A reduction in purine by up to 45%, supplemented with either 0.3% or 0.6% SA, resulted in a decrease in the growth rate of KRC compared to the control group, particularly during the starter and grower periods, but not during the finisher period (42 to 63 d of age). This suggests that the supply of purines from either diet or endogenous sources was insufficient for KRC growth. Although cells can increase endogenous purine synthesis (via *de novo* and salvage pathways) to meet requirements when dietary purines are inadequate, they also have a limited capacity to synthesize purine in the absence of sufficient purine intake [29]. Our findings are consistent with those of Chaiyasit [9], who reported that KRC fed a −30% Pu did not show negative effects on growth performance. A reduction in growth rate was observed only in the starter and grower periods, but not in the finisher period. This could be attributed to the higher growth rate during the early stages of KRC development, which increases the demand for purine. It has been reported that cellular purine requirements increase during periods of rapid growth or limited nutrient intake [30]. In addition, Kubota et al [7] reported that the purine content in the breast muscle of young KRC (14 to 42 d of age) was higher than that in older chickens (56 to 70 d of age), suggesting a heightened rate of purine synthesis during the early stages of muscle development, leading to increased purine requirement. Due to the positive correlation between purine and protein content [31], reducing dietary purine by 30% and 45% resulted in corresponding CP decreases of 1.5 and 3.0%, respectively. While synthetic amino acids were supplemented to meet requirements in all experimental diets, growth performance remained stable at 30% purine reduction but was impaired at 45% reduction. Previous studies suggested that reducing dietary CP by up to 3.0% with synthetic amino acid supplementation maintains broiler growth performance [28], our findings indicate that a 45% purine reduction negatively affected growth despite meeting amino acid requirements. This indicate that factors beyond amino acid adequacy may influence growth when purine levels are substantially reduced. Therefore, to maintain optimal chicken performance, dietary purine reduction should not exceed 30%. Furthermore, while flavonoid supplementation is generally known to enhance growth performance in chickens, our study revealed that supplementing with SA flavonoids had no effect on growth performance at either purine level (−30% and −45% Pu). These findings are consistent with Khimkem [11], who reported no impact on growth performance in KRC from 1 to 63 d of age with SA supplementation at levels of 0.28% to 0.56% in the basal diet.

Interestingly, a reduction in hypoxanthine and total purine contents was observed in the female KRC major breast muscle fed a −30% Pu diet supplemented with 0.6% SA compared to the control group (106.52 vs. 117.29 mg/100 g meat for hypoxanthine and 154.60 vs. 168.45 mg/100 g meat for total purine contents). These results are probably due to the conversion of hypoxanthine into adenine nucleotides, which includes adenosine triphosphate (ATP), adenosine diphosphate (ADP), and adenosine monophosphate (AMP), with ATP serving as the primary form of cellular energy transfer in the skeletal muscle [32]. Based on purine metabolic pathways, dietary-derived hypoxanthine is the only free base that can be reconverted into adenine nucleotides via the salvage pathway by hypoxanthine phosphoribosyl transferase enzyme, which allows its reutilization in the adenine nucleotide pool [8]. In addition, a −30% Pu diet supplemented with 0.6% SA did also not adversely affect the growth performance of KRC, this is likely due to the sufficient purine content in the diet, allowing for adenine nucleotide synthesis to maintain cellular chemical energy transfer (ATP) production in the muscle [32]. Research in mice tissue has indicated that the inhibition of the XO enzyme can lead to the conversion of hypoxanthine to purine nucleotides, such as IMP, via the salvage pathway [33]. Therefore, it is likely that SA flavonoids inhibit this enzyme, promoting the conversion of the remaining hypoxanthine into purine nucleotides. Based on this metabolic pathway, it is suggested that the female KRC group fed the −30% Pu diet supplemented with 0.6% SA could reduce hypoxanthine, as it may be converted into purine nucleotide, thereby balancing the adenine nucleotide pool in the cells via the salvage pathway. Consequently, this dietary intervention did not disrupt cellular homeostasis, resulting in no adverse effects on the growth performance.

Conversely, female KRC fed a −45% Pu diet (supplemented with either 0.3% or 0.6% SA) showed an increase in hypoxanthine and total purine deposition in the meat compared with the −30% Pu group (supplemented with 0.6% SA), which did not differ significantly from the control group. Generally, when purine nucleotides are depleted in muscle cells, the *de novo* purine biosynthesis pathway is upregulated. This results in an increase in the number of intermediate purine derivatives, such as hypoxanthine [32]. This indicates that despite an excessive reduction in dietary purine, muscle cells attempt to balance the purine nucleotide pool to maintain optimal energy levels and cellular homeostasis for various metabolic processes. However, interference with various metabolic pathways, including purine and protein synthesis as well as muscle development [30], may lead to reduced growth in KRC.

Meat quality is a crucial economic factor for animal production. In this study, feeding a Pu diet supplemented with SA did not alter the chemical composition in terms of protein, fat, and ash. In addition, it did not affect meat quality traits such as pH, drip loss, cooking loss, or shear force compared to the control, except for the b* value. The increased b* in the Pu diets can be attributed to the corn gluten meal used in the formulation, which contains high levels of xanthophyll that impart a yellow hue to the meat. This enhanced yellowness color in the meat could actually increase the market value of slow-growing chickens, as yellow-tinted meat is a sensory characteristic strongly preferred by consumers, particularly in traditional markets [34].

In this study, SR-FTIR microspectroscopy was used to investigate the changes in biomolecules in female KRC major breast muscle. Our results show that the KRC fed the −30% Pu diet supplemented 0.3% or 0.6% SA did not show any significant difference in terms of biomolecule composition compared with the control group. Notably, female KRC receiving the −45% Pu diet exhibited major breast muscle with lower lipid content but higher levels of amide I (protein) and glycogen/carbohydrate than those receiving the −30% Pu diet, regardless of supplementation with either 0.3% or 0.6% SA. These findings suggest that different levels of dietary purine significantly alter biomolecules in major breast muscle. Previous studies have demonstrated that dietary nucleotides can directly affect the lipid composition in the breast muscle of broilers by modifying lipid metabolism-related genes and transcription factors [35]. Moreover, under conditions of dietary nucleotide depletion, lipid metabolism, and adipogenesis are disrupted in mice, leading to inhibited lipid storage within adipocytes and the downregulated expression of lipogenic factors [36]. Therefore, the lower lipid content observed in the major breast muscle of KRC fed the −45% Pu in our study may be related to the restriction of dietary purine nucleotides, which could interfere with lipid metabolism and adipogenesis, resulting in reduced lipid deposition in female KRC major breast muscle.

Regarding the glycogen/carbohydrate content in meat, our results indicate that major breast muscle from female KRC fed a −45% Pu had higher levels of carbohydrates, represented by glycogen, compared to meat from KRC fed a −30% Pu and control groups. Glycogen, which is typically stored in the skeletal muscles and liver as an energy reserve, can be derived from intermediates of glucogenic amino acid metabolism, such as alanine, glutamine, threonine, arginine, and valine. These compounds undergo gluconeogenesis to produce glucose, which is subsequently stored in the liver as glycogen [37]. In this study, the higher glycogen content in the meat of the −45% Pu group than in the −30% Pu group was likely due to the activation of liver metabolism. Generally, during periods of low dietary intake, hepatic glycogenolysis serves as the primary source of glucose to maintain homeostasis and meet energy demands, suggesting that chickens degrade liver glycogen before muscle glycogen to meet energy production demands [38].

In our study, SR-FTIR microspectroscopy detected changes in lipid and protein (amide I and amide II) content that were not apparent in the proximate analysis of crude fat and CP content across experimental treatments. This discrepancy can be attributed to SR-FTIR microspectroscopy’s superior sensitivity and specificity compared to traditional proximate analysis methods [39]. SR-FTIR is commonly used to detect molecular structures that are beyond the analytical capabilities of conventional assays [12]. While our results showed that a −45% Pu diet supplemented with SA decrease lipid and increase glycogen/carbohydrate, it also adversely affects growth performance without altering purine content in female KRC major breast muscle. The precise mechanisms by which Pu and SA diets influence meat lipid and glycogen/carbohydrate composition remain unclear and warrant further investigation to understand the mechanisms and metabolic pathways involved in meat quality, particularly purine synthesis. A key limitation of this study was the inability to determine KRC gender at hatching. Although, conventional sexing is possible, this method can increase early chick mortality due to handling-related stress [40]. For the future research, we recommend initiating Pu diet feeding at 21 d of age when sex identification is reliable, and focusing exclusively on female birds to better monitor purine reduction. This modified approach would provide more comprehensive data on purine reduction in female birds while potentially improving economic returns of smallholder farmers.

## CONCLUSION

This study suggests that a 30% reduction in dietary purine, supplemented with 0.6% SA supplementation, effectively decreases purine deposition, particularly hypoxanthine, in the major breast muscle of slow-growing KRC without negative effects on growth performance, meat quality, SUA levels, or the meat chemical and biomolecule composition. However, a 45% reduction in dietary purine impairs growth performance without providing additional benefits to hypoxanthine and purine content, while also altering biomolecule composition through decreased lipids and increased amide I and glycogen/carbohydrates. These findings confirm, for the first time, that the reduction of dietary purines combined with SA flavonoids can decrease purine deposition in chicken meat. Future research should focus on exploring the molecular mechanisms and metabolic pathways to incorporate these insights in improving Pu chicken meat production.

## Figures and Tables

**Table 1:** Composition and nutritional content of the experimental diets (as-fed basis)

Ingredients (%)	Starter (1 to 21 d)					Grower (22 to 42 d)					Finisher (43 to 63 d)				
		
	Control^1)^	−30% Pu^2)^		−45% Pu^3)^		Control	−30% Pu		−45% Pu		Control	−30% Pu		−45% Pu	
					
		0.3% SA	0.6% SA	0.3% SA	0.6% SA		0.3% SA	0.6% SA	0.3% SA	0.6% SA		0.3% SA	0.6% SA	0.3% SA	0.6% SA
Corn	50.18	53.23	52.73	50.76	50.46	54.84	66.23	65.99	69.34	69.16	63.23	72.23	71.63	78.25	77.61
SBM (44% CP)	39.00	17.00	17.00	5.00	5.00	34.60	16.50	16.00	5.00	4.50	27.85	11.77	11.86	3.28	3.40
Rice bran	0.00	4.40	4.40	6.50	6.50	0.00	0.00	0.00	4.00	4.00	0.00	0.00	0.00	0.00	0.00
Rice bran oil	6.38	1.70	1.90	0.00	0.00	6.45	2.18	2.23	0.00	0.00	5.32	1.68	1.89	0.00	0.22
Casava chip	0.00	7.50	7.50	15.00	15.00	0.00	2.00	2.00	4.00	4.00	0.00	3.00	3.00	4.00	4.00
Corn gluten meal	0.00	10.18	10.18	13.30	13.30	0.00	7.38	7.76	10.00	10.37	0.00	6.10	6.10	7.10	7.10
Corn DDGS	0.00	0.00	0.00	2.46	2.46	0.00	0.00	0.00	1.00	1.00	0.00	0.00	0.00	1.30	1.30
SA	0.00	0.30	0.60	0.30	0.60	0.00	0.30	0.60	0.30	0.60	0.00	0.30	0.60	0.30	0.60
Salt	0.48	0.37	0.37	0.33	0.33	0.34	0.26	0.26	0.22	0.22	0.26	0.20	0.20	0.18	0.18
Limestone	1.55	1.61	1.61	1.61	1.61	1.35	1.41	1.41	1.44	1.44	1.26	1.31	1.31	1.32	1.32
MDCP	1.60	1.76	1.76	1.88	1.88	1.53	1.67	1.67	1.74	1.74	1.25	1.40	1.40	1.46	1.46
Premix^4)^	0.50	0.50	0.50	0.50	0.50	0.50	0.50	0.50	0.50	0.50	0.50	0.50	0.50	0.50	0.50
L-Lys HCL	0.00	0.50	0.50	0.80	0.80	0.06	0.51	0.52	0.79	0.80	0.08	0.48	0.48	0.71	0.71
DL-Met	0.27	0.24	0.24	0.24	0.24	0.21	0.19	0.19	0.22	0.21	0.13	0.14	0.14	0.18	0.18
L-Thr	0.04	0.17	0.17	0.27	0.27	0.12	0.24	0.24	0.33	0.33	0.12	0.24	0.24	0.32	0.32
L-Arg	0.00	0.31	0.31	0.55	0.55	0.00	0.30	0.30	0.52	0.53	0.00	0.33	0.33	0.53	0.53
L-Val	0.00	0.08	0.08	0.18	0.18	0.00	0.11	0.11	0.21	0.21	0.00	0.11	0.11	0.21	0.21
L-Trp	0.00	0.07	0.07	0.13	0.13	0.00	0.09	0.09	0.15	0.15	0.00	0.08	0.08	0.13	0.13
L-Ile	0.00	0.08	0.08	0.19	0.19	0.00	0.13	0.13	0.24	0.24	0.00	0.13	0.13	0.23	0.23

1) Control, basal diet.

2) Purine was reduced by 30% from the control diet.

3) Purine was reduced by 45% from the control diet.

4) Premix (5.0 g/kg) provided the following (per kg of diet); 15,000 IU of vitamin A; 3,000 IU of vitamin D3; 25 IU of vitamin E; 5 mg of vitamin K3; 2 mg of vitamin B1; 7 mg of vitamin B2; 4 mg of vitamin B6; 25 mg of vitamin B12; 11.04 mg of pantothenic acid; 35 mg of nicotinic acid; 1 mg of folic acid; 15 μg of biotin; 250 mg of choline chloride; 1.6 mg of Cu; 60 mg of Mn; 45 mg of Zn; 80 mg of Fe; 0.4 mg of I; and 0.15 mg of Se.

Pu, purine; SA, *Sida acuta* Burm. f.; SBM; soybean meal; DDGS, dried distiller’s grains with soluble; MDCP, monocalcium phosphate.

**Table 2:** Calculated and analyzed the chemical composition of experimental diets

Items	Starter (1 to 21 d)					Grower (22 to 42 d)					Finisher (43 to 63 d)				
		
	Control^1)^	−30% Pu^2)^		−45% Pu^3)^		Control	−30% Pu		−45% Pu		Control	−30% Pu		−45% Pu	
					
		0.3% SA	0.6% SA	0.3% SA	0.6% SA		0.3% SA	0.6% SA	0.3% SA	0.6% SA		0.3% SA	0.6% SA	0.3% SA	0.6% SA
Calculated nutrient contents (%)															
Metabolizable energy (kcal/kg)	3,109	3,109	3,109	3,119	3,109	3,175	3,175	3,175	3,175	3,175	3,207	3,207	3,207	3,207	3,207
Crude protein	22.01	20.54	20.50	19.00	18.98	20.45	18.95	18.95	17.45	17.45	18.00	16.50	16.50	15.00	15.00
Calcium	1.00	1.00	1.00	1.00	1.00	0.90	0.90	0.90	0.90	0.90	0.80	0.80	0.80	0.80	0.80
Available P	0.48	0.48	0.48	0.48	0.48	0.46	0.46	0.46	0.46	0.46	0.39	0.39	0.39	0.39	0.39
Digestible Met	0.57	0.57	0.57	0.57	0.57	0.49	0.49	0.49	0.51	0.50	0.39	0.41	0.40	0.43	0.43
Digestible Met + Cys	0.85	0.86	0.86	0.83	0.83	0.75	0.75	0.75	0.75	0.75	0.63	0.63	0.63	0.63	0.63
Digestible Lys	1.08	1.07	1.07	1.07	1.07	1.03	1.03	1.03	1.03	1.03	0.89	0.89	0.89	0.89	0.89
Digestible Thr	0.74	0.74	0.74	0.74	0.74	0.76	0.76	0.76	0.76	0.76	0.68	0.68	0.68	0.68	0.68
Purine (mg/100 g)	125.29	87.39	87.98	68.74	69.42	115.89	81.40	81.11	63.54	63.27	102.03	71.04	71.82	55.69	56.52
Analyzed composition (%)															
Dry matter	91.35	92.11	91.93	92.25	92.21	90.67	91.11	91.30	91.54	91.74	90.12	91.11	91.19	91.37	91.21
Crude protein	22.19	20.75	20.72	19.06	19.83	21.24	19.88	19.70	18.22	18.23	18.10	16.69	16.81	15.12	15.18
Ether extract	8.46	4.69	5.03	3.73	3.59	8.37	4.77	4.85	3.31	3.41	7.70	4.53	4.64	2.96	2.96
Ash	6.88	6.96	7.05	6.91	6.94	6.29	5.90	6.06	6.13	5.55	5.35	5.07	5.21	5.01	5.42
Purine (mg/100 g)	122.65	83.42	81.88	62.07	64.90	116.54	80.49	85.16	75.94	69.24	99.93	71.52	76.43	65.42	60.72

1) Control, basal diet.

2) Purine was reduced by 30% from the control diet.

3) Purine was reduced by 45% from the control diet.

Pu, purine; SA, *Sida acuta* Burm. f.; Met, methionine; Cys, cysteine; Lys, lysine; Thr, threonine.

**Table 3:** Effect of dietary low purine supplemented with *Sida acuta* Burm. f. on growth performance of mixed-sex Korat chickens^1)^

Items	Control^2)^	−30% Pu^3)^		−45% Pu^4)^		SEM	Contrast p-values^5)^			
		
		0.3% SA	0.6% SA	0.3% SA	0.6% SA		Control vs. others	Pu	SA	Pu×SA
Starter (1 to 21 d)										
IBW (g)	42	42	42	42	42	0.059	0.996	0.990	0.970	0.970
BW d 21 (g)	317^a^	306^a^	306^a^	277^b^	280^b^	1.806	<0.001	<0.001	0.730	0.690
BWG (g)	275^a^	264^a^	264^a^	235^b^	238^b^	1.799	<0.001	<0.001	0.728	0.691
FI (g)	422	432	422	418	426	2.160	0.751	0.303	0.755	0.082
FCR (g/g)	1.54^c^	1.64^b^	1.60^bc^	1.78^a^	1.79^a^	0.007	<0.001	<0.001	0.308	0.158
Grower (22 to 42 d)										
BW d 42 (g)	820^a^	792^a^	793^a^	709^b^	720^b^	4.533	<0.001	<0.001	0.532	0.642
BWG (g)	502^a^	486^a^	487^a^	431^b^	440^b^	3.268	<0.001	<0.001	0.498	0.671
FI (g)	1,020	1,011	1,000	1,012	1,031	7.280	0.726	0.339	0.781	0.372
FCR (g/g)	2.03^b^	2.08^b^	2.06^b^	2.34^a^	2.35^a^	0.016	<0.001	<0.001	0.705	0.705
Finisher (43 to 63 d)										
BW d 63 (g)	1,393^a^	1,350^a^	1,347^a^	1,232^b^	1,264^b^	8.634	<0.001	<0.001	0.448	0.367
BWG (g)	574	558	554	523	545	5.468	0.466	0.083	0.495	0.299
FI (g)	1,528	1,518	1,504	1,487	1,517	10.087	0.399	0.706	0.723	0.334
FCR (g/g)	2.67	2.72	2.72	2.84	2.79	0.022	0.078	<0.001	0.499	0.612
Overall (1 to 63 d)										
BWG (g)	1,351^a^	1,307^a^	1,305^a^	1,189^b^	1,222^b^	8.635	<0.001	<0.001	0.447	0.367
FI (g)	2,970	2,960	2,926	2,917	2,974	17.265	0.547	0.956	0.776	0.247
FCR (g/g)	2.20^b^	2.26^b^	2.24^b^	2.45^a^	2.43^a^	0.012	<0.001	<0.001	0.460	0.976

1) The mean values of IBW, BW, BWG, FI and FCR were determined based on mixed-sex birds (6 replicates treatment with 16 birds of each replicate).

2) Control, basal diet.

3) Purine was reduced by 30% from the control diet.

4) Purine was reduced by 45% from the control diet.

5) p-values were obtained by orthogonal contrasts: Control vs. others, control vs. other treatments; Pu level, level of low purine diet; SA level, level of SA supplementation; Pu×SA, the interaction of Pu vs. SA. The probability level of orthogonal contrasts was statistically significant at p<0.05.

a–c Mean values in the same row with different superscripts differ significantly by Tukey’s HSD test at p<0.05.

Pu, purine; SA, *Sida acuta* Burm. f.; SEM, pooled standard error of the means; IBW, initial body weight; BW, body weight; BWG, body weight gain; FI, feed intake; FCR, feed conversion ratio.

**Table 4:** Effect of dietary low purine supplemented with *Sida acuta* Burm. f. on growth performance of female Korat chickens

Items	Control^1)^	−30% Pu^2)^		−45% Pu^3)^		SEM	Contrast p-values^4)^			
		
		0.3% SA	0.6% SA	0.3% SA	0.6% SA		Control vs. others	Pu	SA	Pu×SA
Starter (1 to 21 d)										
IBW (g)	42	43	42	42	42	0.019	0.265	0.427	0.322	0.108
BW d 21 (g)	301^a^	287^a^	293^a^	266^b^	267^b^	1.877	<0.001	<0.001	0.429	0.618
BWG (g)	259^a^	244^a^	250^a^	223^b^	225^b^	1.876	<0.001	<0.001	0.423	0.605
Grower (22 to 42 d)										
BW d 42 (g)	753^a^	708^ab^	729^a^	667^b^	664^b^	5.245	<0.001	<0.001	0.473	0.320
BWG (g)	452^a^	421^ab^	436^a^	401^b^	397^b^	4.006	<0.001	0.001	0.572	0.288
Finisher (43 to 63 d)										
BW d 63 (g)	1,248^a^	1,184^ab^	1,214^ab^	1,145^b^	1,138^b^	8.570	0.003	0.004	0.556	0.357
BWG (g)	496	476	485	477	475	4.529	0.774	0.694	0.774	0.551
Overall (1 to 63 d)										
BWG (g)	1,206^a^	1,141^ab^	1,171^ab^	1,103^b^	1,096^b^	8.571	0.003	0.004	0.554	0.355

1) Control, basal diet.

2) Purine was reduced by 30% from the control diet.

3) Purine was reduced by 45% from the control diet.

4) p-values were obtained by orthogonal contrasts: Control vs. others, control vs. other treatments; Pu level, level of low purine diet; SA level, level of SA supplementation; Pu×SA, the interaction of Pu vs. SA. The probability level of orthogonal contrasts was statistically significant at p<0.05.

The mean values of IBW were determined from mixed-sex birds (6 replicates per treatment with 16 birds per replicate).

a,b Mean values in the same row with different superscripts differ significantly by Tukey’s HSD test at p<0.05.

The mean values of BWG were calculated as the difference between final BW (using only female bird weights) and IBW (derived from mixed-sex birds).

Pu; purine; SA, *Sida acuta* Burm. f.; IBW, initial body weight; BW, body weight; BWG, body weight gain; SEM, pooled standard error of the means.

**Table 5:** Effect of dietary low purine supplemented with *Sida acuta* Burm. f. on major breast muscle quality and chemical composition of female Korat chickens at 63 d of age^1)^

Items	Control^2)^	−30% Pu^2)^		−45% Pu^4)^		SEM	Contrast p-values^5)^			
		
		0.3% SA	0.6% SA	0.3% SA	0.6% SA		Control vs. others	Pu	SA	Pu×SA
Meat quality										
pH_45min_	5.93	5.70	5.90	5.91	5.86	0.027	0.207	0.174	0.218	0.051
pH_24h_	5.78	5.74	5.74	5.80	5.77	0.015	0.615	0.194	0.549	0.649
L* (lightness)	44.59	43.35	44.73	42.19	42.74	0.403	0.195	0.093	0.294	0.649
a* (redness)	0.97	1.98	1.41	2.19	1.66	0.178	0.070	0.573	0.182	0.969
b * (yellowness)	5.77^b^	11.10^a^	11.34^a^	11.78^a^	11.94^a^	0.290	<0.001	0.331	0.762	0.948
Drip loss (%)	6.32	6.87	6.24	6.16	6.31	0.134	0.837	0.297	0.432	0.202
Cooking loss (%)	22.43	21.52	21.87	21.86	21.03	0.218	0.123	0.611	0.633	0.234
Shear force (kg)	2.24	2.47	2.28	2.25	2.36	0.047	0.390	0.514	0.708	0.151
Meat chemical composition (%)										
Dry matter	27.69	27.79	27.64	27.76	27.97	0.081	0.608	0.430	0.891	0.338
Crude protein	26.00	26.36	25.99	26.16	26.36	0.075	0.250	0.637	0.588	0.105
Ether extract	0.30	0.36	0.34	0.36	0.37	0.016	0.140	0.743	1.000	0.674
Ash	1.48	1.50	1.48	1.49	1.51	0.013	0.676	0.887	0.932	0.480

1) Values represent the mean of 6 female birds per treatment group.

2) Control, basal diet.

3) Purine was reduced by 30% from the control diet.

4) Purine was reduced by 45% from the control diet.

5) p-values were obtained by orthogonal contrasts: Control vs. others, control vs. other treatments; Pu level, level of low purine diet; SA level, level of SA supplementation; Pu×SA, the interaction of Pu vs. SA. The probability level of orthogonal contrasts was statistically significant at p<0.05.

a,b Mean values in the same row with different superscripts differ significantly by Tukey’s HSD test at p<0.05.

Pu, purine; SA, *Sida acuta* Burm. f.; SEM, pooled standard error of the means.

**Table 6:** Effect of dietary low purine supplemented with *Sida acuta* Burm. f. on serum uric acid concentration (mg/dL) of female Korat chickens at 21, 42 and 63 d of age^1)^

Age (d)	Control^2)^	−30% Pu^2)^		−45% Pu^4)^		SEM	Contrast p-values^5)^			
		
		0.3% SA	0.6% SA	0.3% SA	0.6% SA		Control vs. others	Pu	SA	Pu×SA
21	11.67	9.00	8.72	9.23	8.10	0.514	0.259	0.869	0.543	0.715
42	7.25^a^	5.72^ab^	5.95^ab^	5.55^ab^	3.48^b^	0.324	0.017	0.081	0.218	0.125
63	7.12	6.30	6.07	6.37	4.92	0.223	0.065	0.288	0.104	0.234

1) Values represent the mean of 6 female birds per treatment group.

2) Control, basal diet.

3) Purine was reduced by 30% from the control diet.

4) Purine was reduced by 45% from the control diet.

5) p-values were obtained by orthogonal contrasts: Control vs. others, control vs. other treatments; Pu level, level of low purine diet; SA level, level of SA supplementation; Pu×SA, the interaction of Pu vs. SA. The probability level of orthogonal contrasts was statistically significant at p<0.05.

a,b Mean values in the same row with different superscripts differ significantly by Tukey’s HSD test at p<0.05.

Pu, purine; SA, *Sida acuta* Burm. f.; SEM, pooled standard error of the means.

**Table 7:** Effect of dietary low purine supplemented with *Sida acuta* Burm. f. on purine and its derivatives deposition in female Korat chickens major breast muscle at 63 d of age^1)^

Items	Control^2)^	−30% Pu^3)^		−45% Pu^4)^		SEM	Contrast p-values^5)^			
		
		0.3% SA	0.6% SA	0.3% SA	0.6% SA		Control vs. others	Pu	SA	Pu×SA
Purine (mg/100 g meat)										
Guanine	27.79	27.24	25.45	27.42	28.53	0.392	0.524	0.074	0.702	0.110
Adenine	23.37	22.08	22.63	24.55	24.47	0.426	0.954	0.291	0.804	0.741
Hypoxanthine	117.29^a^	114.99^ab^	106.52^b^	116.14^a^	118.08^a^	0.952	0.171	0.006	0.138	0.022
Adenine + Hypoxanthine	140.66	137.07	129.15	140.69	142.55	1.095	0.240	0.002	0.228	0.057
Total purine	168.45^a^	164.31^ab^	154.60^b^	168.10^a^	171.08^a^	1.187	0.198	0.001	0.217	0.025
Nucleotide (mg/100 g meat)										
GMP	2.50	2.47	2.47	2.37	2.35	0.055	0.566	0.381	0.962	0.941
IMP	135.24	129.85	147.77	132.44	134.17	4.639	0.944	0.601	0.353	0.443

1) Values represent the mean of 6 female birds replicated per treatment group.

2) Control, basal diet.

3) Purine was reduced by 30% from the control diet.

4) Purine was reduced by 45% from the control diet.

5) p-values were obtained by orthogonal contrasts: Control vs. others, control vs. other treatments; Pu level, level of low purine diet; SA level, level of SA supplementation; Pu×SA, the interaction of Pu vs. SA. The probability level of orthogonal contrasts was statistically significant at p<0.05.

a,b Mean values in the same row with different superscripts differ significantly by Tukey’s HSD test at p<0.05.

Pu, purine; SA, *Sida acuta* Burm. f.; IMP, inosine monophosphate; GMP, guanosine monophosphate; SEM, pooled standard error of the means.

**Table 8:** The ratio of the integral area of biomolecules in female Korat chickens major breast muscle fed low purine diet supplemented with *Sida acuta* Burm. f. was determined using synchrotron radiation-Fourier transform infrared (SR-FTIR) microspectroscopy^1)^

Biomolecules (wavenumber)	% Integral area					SEM	Contrast p-values^5)^			
	
	Control^2)^	− 30% Pu^3)^		−45% Pu^4)^			Control vs. others	Pu	SA	Pu×SA
	
		0.3% SA	0.6% SA	0.3% SA	0.6% SA					
CH stretching of lipids (3,000 to 2,800 cm^−1^)	7.76	8.34	9.57	7.12	7.29	0.216	0.563	0.001	0.162	0.284
Amide I (1,700 to 1,600 cm^−1^)	49.69	47.70	46.38	48.63	48.96	0.364	0.063	0.041	0.547	0.320
Amide II (1,600 to 1,500 cm^−1^)	20.54	21.70	20.76	20.17	20.67	0.209	0.454	0.037	0.987	0.294
CH bending (1,450 to 1,390 cm^−1^)	11.14	10.43	10.35	11.18	10.31	0.153	0.186	0.477	0.297	0.152
Amide III (1,320 to 1,220 cm^−1^)	4.49	4.17	5.17	3.93	4.27	0.148	0.839	0.069	0.039	0.256
Glycogen/Carbohydrate (1,200 to 900 cm^−1^)	6.39^a^	7.66^ab^	6.84^ab^	8.96^b^	8.51^ab^	0.270	0.022	0.028	0.369	0.875

1) Results were averaged from 150 spectra per treatment group.

2) Control, basal diet.

3) Purine was reduced by 30% from the control diet.

4) Purine was reduced by 45% from the control diet.

5) p-values were obtained by orthogonal contrasts: Control vs. others, control vs. other treatments; Pu level, level of low purine diet; SA level, level of SA supplementation; Pu×SA, the interaction of Pu vs. SA. The probability level of orthogonal contrasts was statistically significant at p<0.05.

Pu, purine; SA, *Sida acuta* Burm. f.; SEM, pooled standard error of the means.

**Table 9:** The relative proportion (%) of secondary protein structures in the amide I region of female Korat chickens major breast muscle fed low purine diet supplemented with *Sida acuta* Burm. f. using synchrotron radiation-Fourier transform infrared (SR-FTIR) microspectroscopy^1)^

Biomolecules (wavenumber)	% Integral area					SEM	Contrast p-values^5)^			
	
	Control^2)^	− 30% Pu^3)^		−45% Pu^4)^			Control vs. others	Pu	SA	Pu×SA
	
		0.3% SA	0.6% SA	0.3% SA	0.6% SA					
β-sheet (1,623 cm^−1^, 1,635 cm^−1^)	26.87	24.58	26.37	22.90	25.19	0.532	0.126	0.241	0.099	0.836
α-helix (1,650 cm^−1^, 1,660 cm^−1^)	45.12	45.03	44.20	47.88	46.02	0.810	0.748	0.209	0.465	0.779
β-turn (1,675 cm^−1^)	16.58	16.95	17.13	16.74	15.45	0.654	0.996	0.523	0.708	0.619
Antiparallel (1,690 cm^−1^)	11.43	13.43	12.30	12.48	13.33	0.466	0.224	0.970	0.895	0.346

1) Results were averaged from 150 spectra per treatment group.

2) Control, basal diet.

3) Purine was reduced by 30% from the control diet.

4) Purine was reduced by 45% from the control diet.

5) p-values were obtained by orthogonal contrasts: Control vs. others, control vs. other treatments; Pu level, level of low purine diet; SA level, level of SA supplementation; Pu×SA, the interaction of Pu vs. SA. The probability level of orthogonal contrasts was statistically significant at p<0.05.

Pu, purine; SA, *Sida acuta* Burm. f.; SEM, pooled standard error of the means.

## References

[1] Zhang Y, Chen S, Yuan M, Xu Y, Xu H (2022). Gout and diet: a comprehensive review of mechanisms and management. Nutrients.

[2] Chen Z, Xue X, Ma L (2024). Effect of low-purine diet on the serum uric acid of gout patients in different clinical subtypes: a prospective cohort study. Eur J Med Res.

[3] Tran DH, Schonewille JT, Pukkung C, Khempaka S (2021). Growth performance and accretion of selected amino acids in response to three levels of dietary lysine fed to fast- and slow-growing broilers. Poult Sci.

[4] Kiratikrankul B, Yongsawatdigul J Purines and meat quality of chicken meat from various breeds.

[5] Munyaneza JP, Kim M, Cho E, Jang A, Choo HJ, Lee JH (2023). Association of single-nucleotide polymorphisms in dual specificity phosphatase 8 and insulin-like growth factor 2 genes with inosine-5,-monophosphate, inosine, and hypoxanthine contents in chickens. Anim Biosci.

[6] Jantasaeng O, Duclos MJ, Khempaka S (2024). Effects of feeding strategies to reduce purine content in meat from slow-growing Korat chickens. Anim Sci Proc.

[7] Kubota S, Vandee A, Keawnakient P, Molee W, Yongsawatdikul J, Molee A (2019). Effects of the MC4R, CAPN1, and ADSL genes on body weight and purine content in slow-growing chickens. Poult Sci.

[8] Camici M, Garcia-Gil M, Allegrini S (2023). Inborn errors of purine salvage and catabolism. Metabolites.

[9] Chaiyasit S (2019). Effect of low purine diets on growth performance, meat quality and purine accumulation in Korat chicken meat [master’s thesis].

[10] Ogunmoyole T, Falusi OO, Oderinde F (2022). Sida acuta leaf extract attenuates oxidants-induced animal model of nephrotoxicity and hepatotoxicity. Clin Phytosci.

[11] Khimkem A (2018). Effect of flavonoid and hypoxanthine phosphoribosyl transferase enzyme on growth performance, meat quality and purine accumulation in chicken meat [master’s thesis].

[12] Yu P (2004). Application of advanced synchrotron radiation-based Fourier transform infrared (SR-FTIR) microspectroscopy to animal nutrition and feed science: a novel approach. Br J Nutr.

[13] Katemala S, Molee A, Thumanu K, Yongsawatdigul J (2022). A comparative study of meat quality and vibrational spectroscopic properties of different chicken breeds. Poult Sci.

[14] National Research Council (NRC) (1994). Nutrient requirements of poultry.

[15] Maliwan P, Khempaka S, Molee W, Schonewille JT (2018). Effect of energy density of diet on growth performance of Thai indigenous (50% crossbred) Korat chickens from hatch to 42 days of age. Trop Anim Health Prod.

[16] Maliwan P, Molee W, Khempaka S (2019). Response of Thai indigenous crossbred chickens to various dietary protein levels at different ages. Trop Anim Health Prod.

[17] Ajinomoto Heartland LLC (2004). True digestibility of essential amino acids for poultry.

[18] Daneshmand A, Kermanshahi H, Danesh Mesgaran M, King AJ, Ibrahim SA (2017). Effect of purine nucleosides on growth performance, gut morphology, digestive enzymes, serum profile and immune response in broiler chickens. Br Poult Sci.

[19] Chen X, Jiang W, Tan HZ (2013). Effects of outdoor access on growth performance, carcass composition, and meat characteristics of broiler chickens. Poult Sci.

[20] Molee W, Khosinklang W, Tongduang P, Thumanu K, Yongsawatdigul J, Molee A (2022). Biomolecules, fatty acids, meat quality, and growth performance of slow-growing chickens in an organic raising system. Animals.

[21] Association of Official Analytical Chemists (AOAC) (1990). Official methods of analysis.

[22] Association of Official Analytical Chemists (AOAC) (2006). Official methods of analysis of AOAC International.

[23] Kaneko K, Aoyagi Y, Fukuuchi T, Inazawa K, Yamaoka N (2014). Total purine and purine base content of common foodstuffs for facilitating nutritional therapy for gout and hyperuricemia. Biol Pharm Bull.

[24] Fossati P, Prencipe L, Berti G (1980). Use of 3,5-dichloro-2-hydroxybenzenesulfonic acid/4-aminophenazone chromogenic system in direct enzymic assay of uric acid in serum and urine. Clin Chem.

[25] Suwanvichanee C, Sinpru P, Promkhun K (2022). Effects of β-alanine and L-histidine supplementation on carnosine contents in and quality and secondary structure of proteins in slow-growing Korat chicken meat. Poult Sci.

[26] Tantiyasawasdikul V, Chomchuen K, Loengbudnark W, Chankitisakul V, Boonkum W (2023). Comparative study and relationship analysis between purine content, uric acid, superoxide dismutase, and growth traits in purebred and crossbred Thai native chickens. Front Vet Sci.

[27] Katemala S, Molee A, Thumanu K, Yongsawatdigul J (2021). Meat quality and Raman spectroscopic characterization of Korat hybrid chicken obtained from various rearing periods. Poult Sci.

[28] Hernández F, López M, Martínez S, Megías MD, Catalá P, Madrid J (2012). Effect of low-protein diets and single sex on production performance, plasma metabolites, digestibility, and nitrogen excretion in 1- to 48-day-old broilers. Poult Sci.

[29] Sánchez-Pozo A, Gil A (2002). Nucleotides as semiessential nutritional components. Br J Nutr.

[30] Mohamed FF, Hady MM, Kamel NF, Ragaa NM (2020). The impact of exogenous dietary nucleotides in ameliorating Clostridium perfringens infection and improving intestinal barriers gene expression in broiler chicken. Vet Anim Sci.

[31] Havlik J, Plachy V, Fernandez J, Rada V (2010). Dietary purines in vegetarian meat analogues. J Sci Food Agric.

[32] Tomczyk M, Glaser T, Slominska EM, Ulrich H, Smolenski RT (2021). Purine nucleotides metabolism and signaling in Huntingtonnucle. Purine nucleoor a target for novel therapies. Int J Mol Sci.

[33] Hosoya T, Uchida S, Shibata S, Tomioka NH, Matsumoto K, Hosoyamada M (2022). Xanthine oxidoreductase inhibitors suppress the onset of exercise-induced AKI in high HPRT activity Urat1-Uox double knockout mice. J Am Soc Nephrol.

[34] Sow TMA, Grongnet JF (2010). Sensory characteristics and consumer preference for chicken meat in Guinea. Poult Sci.

[35] Tansutaphanit S, Haga Y, Kabeya N (2023). Impact of purine nucleotide on fatty acid metabolism and expression of lipid metabolism-related gene in the liver cell of rainbow trout Oncorhynchus mykiss. Comp Biochem Physiol B Biochem Mol Biol.

[36] Shinde AB, Nunn ER, Wilson GA (2023). Inhibition of nucleotide biosynthesis disrupts lipid accumulation and adipogenesis. J Biol Chem.

[37] Métayer-Coustard S, Tesseraud S, Praud C (2021). Early growth and protein-energy metabolism in chicken lines divergently selected on ultimate pH. Front Physiol.

[38] Zhang X, Yang S, Chen J, Su Z (2019). Unraveling the regulation of hepatic gluconeogenesis. Front Endocrinol.

[39] Al-Bakri AG, Dahabiyeh LA, Khalil E (2022). Synchrotron-radiation-based Fourier transform infrared microspectroscopy as a tool for the differentiation between staphylococcal small colony variants. Antibiotics.

[40] England AD, Kheravii SK, Musigwa S (2021). Sexing chickens (Gallus gallus domesticus) with high-resolution melting analysis using feather crude DNA. Poult Sci.

